# Contact heat sensitivity and reports of unpleasantness in communicative people with mild to moderate cognitive impairment in Alzheimer’s disease: a cross-sectional study

**DOI:** 10.1186/s12916-016-0619-1

**Published:** 2016-05-10

**Authors:** Todd B. Monroe, Stephen J. Gibson, Stephen P. Bruehl, John C. Gore, Mary S. Dietrich, Paul Newhouse, Sebastian Atalla, Ronald L. Cowan

**Affiliations:** Vanderbilt University School of Nursing, Vanderbilt University Institute of Imaging Science, Vanderbilt Psychiatric Neuroimaging Program, Nashville, Tennessee USA; National Ageing Research Institute, Royal Melbourne Hospital, PO Box 2127, Melbourne, VIC 3050 Australia; Vanderbilt University School of Medicine, Nashville, Tennessee USA; Vanderbilt University Institute of Imaging Science, Nashville, Tennessee USA; Vanderbilt Psychiatric Neuroimaging Program, Vanderbilt University Schools of Medicine and Nursing, Nashville, Tennessee USA; Vanderbilt Center for Cognitive Medicine, Vanderbilt University School of Medicine, Nashville, Tennessee USA; Vanderbilt Psychiatric Neuroimaging Program, Vanderbilt University School of Medicine, Nashville, Tennessee USA

**Keywords:** Pain, Dementia, Alzheimer’s disease, Unpleasantness, Psychophysics

## Abstract

**Background:**

Compared to healthy controls, people with Alzheimer’s disease (AD) have been shown to receive less pain medication and report pain less frequently. It is unknown if these findings reflect less perceived pain in AD, an inability to recognize pain, or an inability to communicate pain.

**Methods:**

To further examine aspects of pain processing in AD, we conducted a cross-sectional study of sex-matched adults ≥65 years old with and without AD (AD: *n* = 40, female = 20, median age = 75; control: *n* = 40, female = 20, median age = 70) to compare the psychophysical response to contact-evoked perceptual heat thresholds of warmth, mild pain, and moderate pain, and self-reported unpleasantness for each percept.

**Results:**

When compared to controls, participants with AD required higher temperatures to report sensing warmth (Cohen’s *d* = 0.64, *p* = 0.002), mild pain (Cohen’s *d* = 0.51, *p* = 0.016), and moderate pain (Cohen’s *d* = 0.45, *p* = 0.043). Conversely, there were no significant between-group differences in unpleasantness ratings (*p* > 0.05).

**Conclusions:**

The between-group findings demonstrate that when compared to controls, people with AD are less sensitive to the detection of thermal pain but do not differ in affective response to the unpleasant aspects of thermal pain. These findings suggest that people with AD may experience greater levels of pain and potentially greater levels of tissue or organ damage prior to identifying and reporting injury. This finding may help to explain the decreased frequency of pain reports and consequently a lower administration of analgesics in AD.

**Electronic supplementary material:**

The online version of this article (doi:10.1186/s12916-016-0619-1) contains supplementary material, which is available to authorized users.

## Background

Poorly managed pain in people with Alzheimer’s disease (AD) is a significant public health concern. AD in general is a risk factor for the under-treatment of pain, due in part to a lack of understanding of the impact of AD on psychophysiological factors that influence the pain experience. In the presence of similar painful conditions, when compared to cognitively intact older adults, people with AD have been shown to receive less pain medication [1–3]. However, a recent large-scale study found that when compared to people without dementia, people with dementia reported pain less frequently and although they reportedly used acetaminophen more frequently, there were no significant differences in the use of opioids and NSAIDs [4]. Furthermore, in the presence of similar painful conditions, people with AD verbally report pain less frequently [5, 6] but exhibit similar pain-related behaviors when moved [6]. It is unknown if these findings reflect less perceived pain in AD or an inability to recognize pain or to communicate pain.

**Fig. 1:**
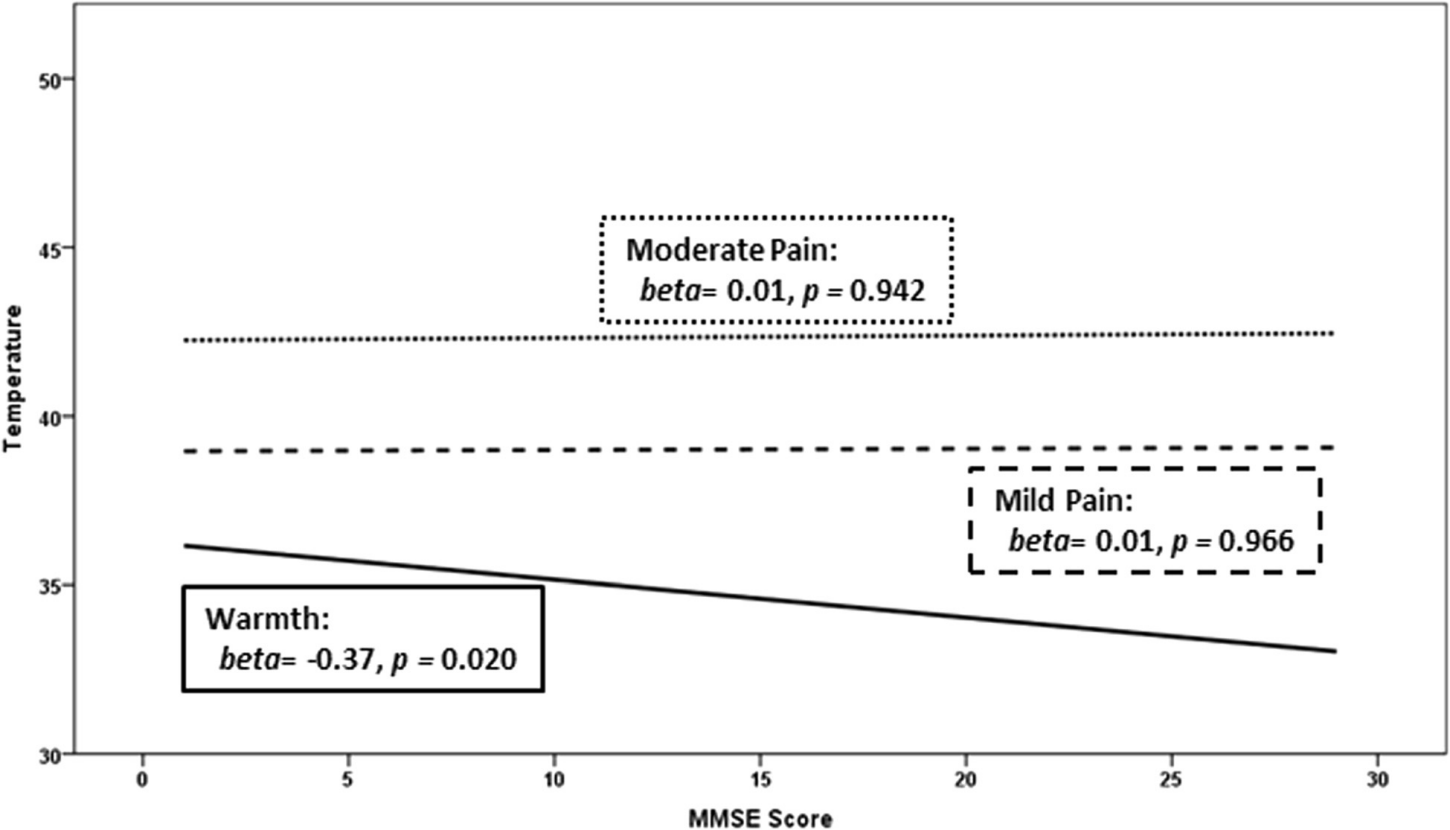
Within the Alzheimer’s disease (AD) group only, association of temperature sensation for the detection of “warmth,” “mild pain,” and “moderate pain” in degrees Celsius (range = 30–55 degrees Celsius) and global cognitive impairment in AD (Mini-Mental State Examination [*MMSE*] score; range 0 = completely cognitively impaired to 30 = completely cognitively intact)

**Fig. 2:**
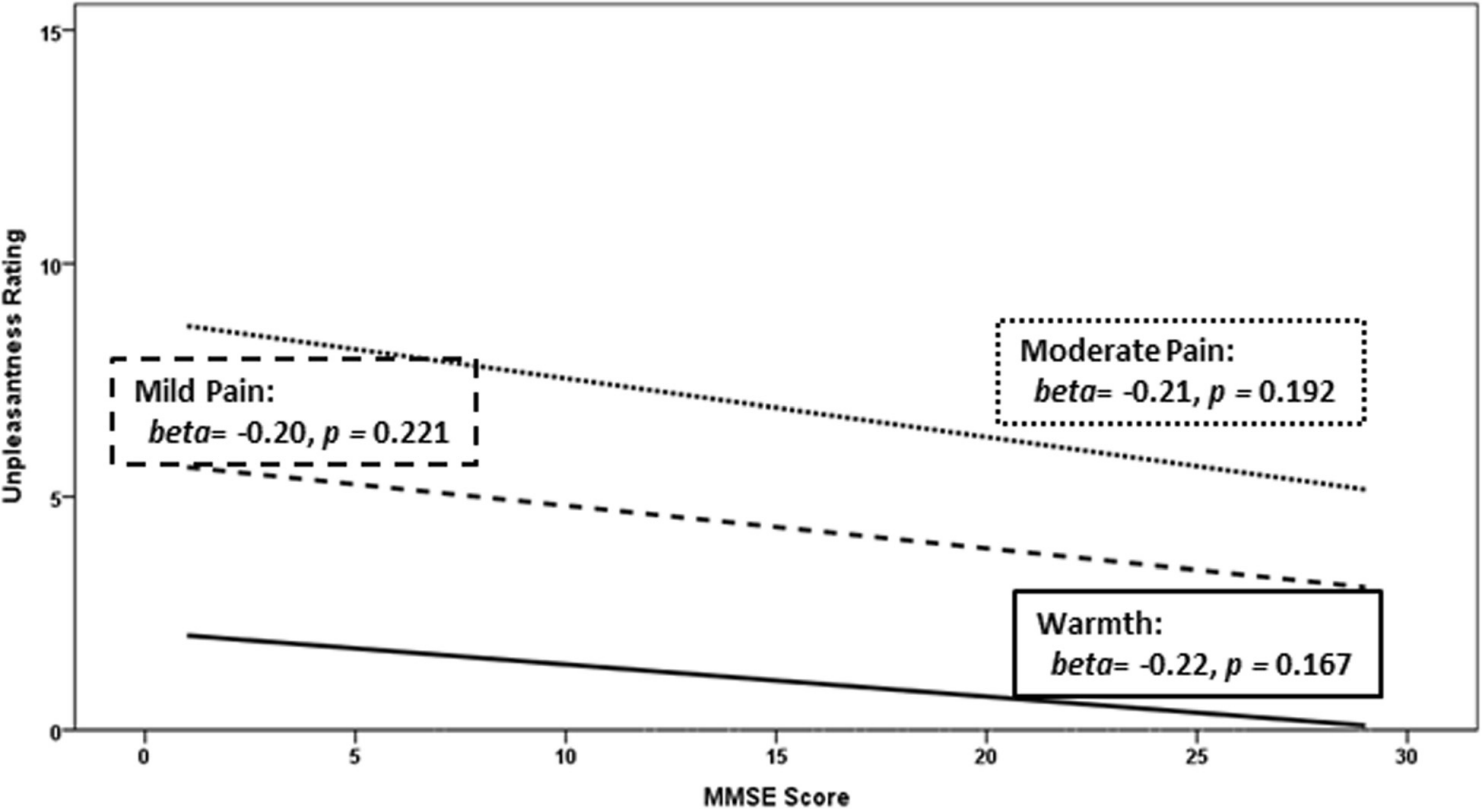
Within the Alzheimer’s disease (AD) group only, association of unpleasantness ratings (range 0 = neutral to 20 = extremely intolerable) for “warmth,” “mild pain,” and “moderate pain” and global cognitive impairment in AD (Mini-Mental State Examination [*MMSE*] score; range 0 = completely cognitively impaired to 30 = completely cognitively intact)

Nociceptive studies of pain examine underlying neurophysiological processes that may lead to the perception of pain [7] whereas psychophysical studies examine self-reports of pain perception [8]. This paper will examine the latter. Pain is described as a multidimensional experience consisting of sensory, cognitive, and affective components [9]. If one or more of these components is altered in AD, the ability to detect and report pain may also be altered. Findings from acute experimental pain studies in AD are mixed. In response to contact heat stimuli [10, 11], experimental electrical shock [12, 13], mechanical pressure [14], CO_2_ laser [15], and ischemia [16], the self-reported pain threshold does not differ between healthy controls and people with AD. However, the magnitude of stimulus intensity required to reach tolerance (stimulus reported as “unbearable”) was significantly higher in AD, with pain tolerance increasing as AD severity worsened [16]. Conversely, for suprathreshold heat pain stimuli, people with AD reported more pain (increased sensitivity) when compared to controls [11]. Relative to controls, people with AD demonstrated a blunted autonomic response yet normal pain perception to a painful stimulus just above the pain threshold; however, when the pain stimulus was increased to twice the threshold, people with AD demonstrated a blunted perceptual response to pain (but no difference in autonomic response) [10]. These findings suggest that people with AD have a variable pain response that is threshold dependent [12]. Gibson and colleagues found that when compared to controls, individuals with AD demonstrated increased detection thresholds for just-noticeable sensation but the groups did not differ in their intensity ratings in response to fixed temperatures [15]. Cole and colleagues found that compared to healthy controls, people with AD displayed increased mechanical pressure pain thresholds (i.e., decreased sensory sensitivity) for just-noticeable pain and weak pain while also reporting just-noticeable pain as more unpleasant [17].

As an alternative to examining verbal reports of pain, some laboratories are examining facial affective responses to pain, demonstrating that facial coding can effectively be used to measure affective responses to evoked pain (presumably corresponding to pain unpleasantness) in people with AD [18]. Kunz and colleagues observed that, relative to controls, people with dementia demonstrated increased facial pain affect to both mechanical pressure and electrical shock [5, 14]. Similarly, Beach and colleagues recently found that in response to pressure algometry, people with AD demonstrated increased facial pain affect when compared to controls [19].

In summary, these experimental pain studies suggest that AD is variously associated with increased pain threshold and tolerance with diminished sensitivity, no differences relative to healthy individuals, or even increased pain unpleasantness. Reconciling these variations across studies is difficult, but possible reasons include the use of different experimental pain stimuli, different measures of psychophysics, varying degrees of cognitive impairment, and limited sample sizes.

Though the above-mentioned studies have provided important insight into our understanding of pain processing in dementia, no consensus has yet been reached regarding the impact of AD on the experience of pain. Reasons for this lack of consensus include the following: (1) measuring stimulus intensity threshold verses pain tolerance, (2) few studies have examined both threshold intensity and self-report of pain, (3) few studies have examined suprathreshold intensities, and (4) few studies have examined suprathreshold stimuli which may be more relevant to clinical pain when compared to pain threshold. Nonetheless, there are efforts being made to reconcile this literature. A recent review of responses to experimental pain stimuli found that people with cognitive impairment generally exhibit hypersensitivity rather than hyposensitivity to pain [20]. Despite these efforts, key clinical questions remain unanswered. The most important is whether decreased treatment of pain in AD results from a decreased ability to recognize and report pain, or whether AD-related changes in pain systems result in altered pain perception relative to healthy individuals. Such questions must be addressed to help inform future research endeavors that seek to develop evidence-based pain management in AD.

The purpose of this cross-sectional study was to compare the psychophysical response to experimental thermal pain in a group of older adults with AD to comparable individuals without AD. To our knowledge, this is the first study to use experimental thermal stimuli to assess pain perception at suprathreshold levels in conjunction with verbal reports of unpleasantness in people diagnosed with mild to moderate cognitive impairment in AD. Our first hypothesis was that, when compared to sex and age-matched cognitively intact controls, people with AD would be less sensitive to thermal pain and find pain to be less unpleasant. Our second hypothesis was that, among people with AD, a surrogate index of greater AD severity, namely an increasing global cognitive impairment as measured by the Folstein Mini-Mental State Examination (MMSE) [21], would be associated with higher thresholds for detecting thermal stimuli (at warmth, mild pain, and moderate pain intensities) and lower reported unpleasantness associated with each of these intensities.

## Methods

### Participants

From a sample of 97 people ≥65 years old completing a larger study of mechanisms underlying AD-related alterations in pain responsiveness, 40 older adults with AD were matched to 40 older adults without AD. The samples had equal sex distributions (female = 20, male = 20) and very similar age distributions (control: median = 70 years; AD: median = 75 years). Four research assistants enrolled the participants included in the current study over a 3-year period from 2012 to 2015. Participants with a clinical diagnosis of AD were recruited from the practices of three geriatricians, two geriatric psychiatrists, and a neurologist from Vanderbilt University Medical Center. Medical records were reviewed to confirm the presence of an AD diagnosis based on supportive documentation including the following: (1) neuropsychiatric evaluation, diagnostic MRI or PET, and/or serum laboratory tests eliminating other potential causes of memory loss (e.g., vitamin B12, thyroid hormone, complete blood count, electrolyte balance, HIV), and (2) clinical diagnostic support based on results on evaluation with the MMSE [21], the Montreal Cognitive Assessment (MoCA) [22], and/or the Functional Assessment Staging (FAST) Scale [23]. Although not all measures necessary to evaluate the criteria were consistently available in the medical records, approximately 62 % of patients met the National Institute of Neurological and Communicative Disorders and Stroke and the Disease and Related Disorders Association (NINCDS-ADRDA) [24] criteria for AD. The extent of global cognitive impairment in people with AD was assessed with the MMSE. To be included, all participants with AD were required to be verbally communicative and able to provide a verbal pain rating. Controls were recruited from Vanderbilt University’s Research Match Trial Registry and via advertising through posted flyers from the greater Nashville, TN, USA, metropolitan area.

To reduce potential confounds to testing study hypotheses and for safety reasons, potential participants were excluded for regular use of opioid or non-opioid pain medications, history of stroke, cancer, peripheral neuropathy, unstable cardiac conditions, unstable respiratory conditions, insulin-dependent diabetes, psychiatric disorders (e.g., schizophrenia, bipolar disorder, depression), current or recent substance use disorders, or Parkinson’s disease. Using these exclusion criteria resulted in a physiologically healthy group of people clinically diagnosed with AD. With their legal surrogate present when appropriate (i.e., for individuals with AD), participants were instructed to avoid taking any pain medication (opioid or non-opioid) for at least 24 hours prior to data collection. The current study was approved by the Vanderbilt University Institutional Review Board. Prior to enrollment, each participant provided written informed consent. In individuals lacking decisional capacity, participant assent and legal surrogate consent were obtained. Participants and their caregivers were each reimbursed US$100 for their time.

### Procedures

#### Measures

Prior to the collection of any questionnaire data and pain psychophysics, participants (and their legal caregiver when present) were presented with an opportunity to experience the thermal pain stimulus and to complete two practice psychophysics trials. Practice trials were completed prior to psychophysical testing to ensure participants understood the directions and that their responses were appropriate (e.g., that warmth detection occurred at a lower temperature than mild pain detection, which occurred at a lower temperature than moderate pain detection).

All study measures were collected at the participant’s residence or Vanderbilt University Medical Center. After meeting the initial screening requirements above, and before continuing with enrollment, each participant’s capacity to consent was assessed using the University of California San Diego Brief Assessment of Capacity to Consent (UBACC) [25]. This form takes less than 5 minutes to complete, with scores >14.5 being 89 % sensitive and 100 % specific for determining capacity to consent for research [25]. Participants with scores >14.5 on the UBACC were permitted to sign the informed consent document; individuals with scores ≤14.5 were permitted to sign the assent document and legal surrogate consent was obtained.

After obtaining consent, participants underwent 1 hour of demographic and psychosocial assessments. Because the current study included communicative people with mild to moderate cognitive impairment in AD, a trained research assistant administered all demographic and data questionnaires orally to all participants. These included a detailed list of all medications and the Hollingshead Four-Factor Index of Socioeconomic Status (SES) [26]. The Brief Pain Inventory Short Form (BPI-SF) was used to collect current clinical pain and average daily pain [27]. Depression and anxiety screens included the Geriatric Depression Scale Short Form (GDS-SF) [28] and the state and trait forms of the Spielberger State-Trait Anxiety Inventory [29], respectively.

### Psychophysical thermal stimulation protocol

The psychophysical testing procedures used in this study have been previously described in detail [30]. In brief, responses to thermal stimuli were assessed with the Medoc Q-Sense (Medoc Ltd., Rimat Yishai, Israel) or the Medoc fMRI-compatible ATS-CHEPS Model Pain and Sensory Evaluation System (Medoc Ltd.) [31]. Both systems were calibrated and set to deliver a stimulus intensity beginning at a baseline of 30 °C with an upward ramp rate of 1 °C/s using a 30 mm × 30 mm thermode placed on the thenar eminence of the right hand. We modified the stimulus intensity matching the protocol used by Cole and colleagues in their successful psychophysical study of mechanical pressure pain in people with AD [17]. Before beginning, it was explained to participants that there were two qualities of pain they would be asked to report: the intensity, that is “how strong the pain feels,” and the unpleasantness, “how unpleasant or disturbing the pain is for you” [32]. Next the Method of Limits program was used to deliver a thermal stimulus. Participants were instructed to press a button to stop the heat stimulus when they perceived (in separate trials) the sensations of warmth, mild pain, and moderate pain. Participants were then told, “After you stop the heat, I will ask you to tell me how unpleasant the previous temperature was.” To assess unpleasantness, participants were then shown a 0–20 unpleasantness scale (0 = neutral, 20 = very intolerable) that has been successfully used in research in older adults with AD [17, 33–35]. Thermal paradigm instructions were repeated before each thermal stimulus delivery and then the instructions for rating unpleasantness were repeated after each stimulus delivery. After completing practice trials, three trials of each of the three stimulus conditions and associated unpleasantness ratings were conducted, and the average temperature and unpleasantness rating for each percept (warmth, mild pain, and moderate pain) were recorded.

### Data analysis

There were no missing values for the critical study variables (AD diagnosis, sex, age, BPI-SF scores). Missing data for the other demographic and sample descriptor variables were random in nature. The continuous demographic, standardized measures, and psychophysical values were summarized using median and inter-quartile range (IQR) due to lack of normality (Fisher test > ±2.58, *p* < 0.01). Categorical data were summarized using frequency distributions. Comparisons between the AD group and the control group were conducted using nonparametric Mann–Whitney tests (continuous and ordinal data) and chi-square tests (categorical data). Generalized linear modeling using the log link function was used to test for main and interaction effects of AD and sensory level (i.e., warmth, mild pain, or moderate pain) intensity on the temperatures and ratings of unpleasantness. Cohen’s *d* effect size indices were generated to summarize the magnitude of the AD effects. Linear regressions within each sensory level using rank-transformed data were used to assess the associations of the severity of global cognitive impairment (MMSE scores) with temperature thresholds and unpleasantness ratings. Due to the known associations of depression with perceptions of pain intensity, the primary analyses were repeated with GDS-SF scores included as covariates. An alpha of *p* < 0.05 was used for determining statistical significance.

## Results

### Demographics

Statistically significant differences between the AD and control groups were observed for SES, which were due to differences in education level (*p* = 0.044). Participants in the control group tended to be more highly educated than those in the group with AD. No other statistically significant demographic differences were observed other than for the two variables known to be associated with cognitive decline (MMSE and GDS-SF depressive symptoms, both *p* < 0.001; see Table 1).

**Table 1:** Demographic and clinical summaries by Alzheimer’s diagnosis

	Total (*N* = 80)	Control (*N* = 40)	AD (*N* = 40)	*p*-value
	N (%)	N (%)	N (%)	
Gender				1.000
Female	40 (50.0)	20 (50.0)	20 (50.0)	
Male	40 (50.0)	20 (50.0)	20 (50.0)	
Race	*N* = 79	*n* = 39	*n* = 40	0.818
African American	10 (12.7)	4 (10.3)	6 (15.0)	
Asian	2 (2.5)	1 (2.6)	1 (2.5)	
Caucasian	67 (84.8)	34 (87.2)	33 (82.5)	
Education	*N* = 77	*n* = 38	*n* = 39	0.044
High school or less	14 (18.2)	5 (13.2)	9 (23.1)	
Partial college	20 (26.0)	11 (28.9)	9 (23.1)	
College graduate	22 (28.6)	7 (18.4)	15 (38.5)	
Graduate school	21 (27.3)	15 (39.5)	6 (15.4)	
Marital status	*N* = 78	*n* = 39	*n* = 39	0.845
Single/divorced/separated	16 (20.5)	9 (23.1)	7 (17.9)	
Married	46 (59.0)	22 (56.4)	24 (61.5)	
Widowed	16 (20.5)	8 (20.5)	8 (20.5)	
Marriage occupation status	*N* = 77	*n* = 39	*n* = 38	0.736
One spouse employed	40 (51.9)	21 (53.8)	19 (50.0)	
Both employed	37 (48.1)	18 (46.2)	19 (50.0)	
	*N*, median [IQR]	*N*, median [IQR]	*N*, median [IQR]	
Spouse’s occupation score	78, 3.5 [0–7]	39, 3.0 [0–8]	39, 4.0 [0–7]	0.567
Age	80, 73.0 [68–80]	40, 70.0 [66–81]	40, 75.0 [71–79]	0.089
Standardized measures				
Body mass index	77, 25.5 [23–29]	40, 25.5 [23–29]	37, 25.1 [22–28]	0.269
Total SES score^a^	77, 55.0 [19–30]	39, 57.0 [45–64]	38, 50.0 [39–56]	0.046
MMSE score^b^	79, 27.0 [19–30]	39, 30.0 [29–30]	40, 19.5 [14–24]	<0.001
BPI-SF average pain^c^	80, 0.0 [0–2]	40, 1.0 [0–2]	40, 0.0 [0–2]	0.059
BPI-SF pain right now^c^	80, 0.0 [0–0]	40, 0.0 [0–1]	40, 0.0 [0–0]	0.203
GDS-SF score^d^	78, 1.0 [0–4]	40, 0.0 [0–2]	38, 3.0 [1–5]	<0.001
STAI state score^e^	68, 47.0 [44–50]	38, 47.0 [44–50]	30, 47.0 [43–50]	0.813
STAI trait score^e^	68, 48.0 [45–51]	38, 48.5 [45–53]	30, 47.0 [44–51]	0.230

^a^ *SES* Hollingshead Four Factor Index of Socioeconomic Status (range = 8–66; 8 = lowest, 66 = highest)

^b^ *MMSE* Folstein Mini Mental State Examination (range = 0–30; 0 = completely cognitively impaired, 30 = completely cognitively intact)

^c^ *BPI-SF* Brief Pain Inventory Short Form (range = 0–10; 0 = no pain, 10 = most pain)

^d^ *GDS-SF* Geriatric Depression Scale Short Form (range = 0–15; 0 = no indication of depression, 15 = high possibility of depression)

^e^ *STAI* Spielberger State or Trait Anxiety Inventory (range 20–80; 20 = indicates increased anxiety, 80 = indicates least amount of anxiety)

*AD* Alzheimer’s disease, *IQR* interquartile range

### Psychophysical results

#### Between-groups effects

A statistically significant main effect of AD group versus control was observed for the stimulus intensity required to evoke the three targeted sensory descriptors (Wald chi-square _(df = 1)_ = 7.71, *p* = 0.005). Pairwise comparisons indicated that participants with AD required higher temperatures to report sensing “warmth” (Cohen’s *d* = 0.64, *p* = 0.002), “mild pain” (Cohen’s *d* = 0.51, *p* = 0.016), and “moderate pain” (Cohen’s *d* = 0.45, *p* = 0.043; see Table 2). There were no statistically significant differences between the groups in ratings of unpleasantness at any stimulus intensity (Wald chi-square _(df = 1)_ = 0.05, Cohen’s *d* = 0.04–0.18, *p* = 0.823; see Table 2). Similar findings for both temperature and unpleasantness were observed from additional analyses that controlled for GDS-SF (depressive symptom) scores.

**Table 2:** Psychophysical summary of temperature thresholds necessary to produce ratings at each condition in a sex-age matched sample of people with and without Alzheimer’s disease

	Total (*N* = 80)	Control (*N* = 40)	AD (*N* = 40)	*p*-value	Cohen’s *d*
	Median [IQR]	Median [IQR]	Median [IQR]		
Just-noticeable warmth					
Temperature	33.0 [32–35]	32.0 [32–34]	34.0 [33–36]	0.002	0.64
Affect	0.0 [0–2]	0.0 [0–2]	0.0 [0–1]	0.846	0.04
Weak pain					
Temperature	37.0 [35–41]	35.5 [34–39]	39.0 [35–42]	0.016	0.51
Affect	4.0 [0–5]	3.3 [0–5]	4.0 [1–6]	0.402	0.18
Moderate pain					
Temperature	42.0 [38–45]	40.0 [38–44]	43.0 [39–45]	0.043	0.45
Affect	6.0 [5–8]	6.0 [5–8]	6.5 [4–9]	0.867	0.04

Temperature was measured in degrees Celsius (range = 30–55 °C). Affect is the affective distress measured via a 0–20 Unpleasantness scale (0 = neutral, 20 = extremely unpleasant). The *p*-value was derived from a Mann–Whitney U Test. *AD* Alzheimer’s disease, *IQR* interquartile range

#### Effects of severity of global cognitive impairment

Hypotheses regarding effects of worsening global cognitive impairment on pain responses were tested within the AD group alone (because there was minimal variation in MMSE in the control group). Results revealed that worsening global cognitive ability was associated with higher temperatures required to report “warmth” (*beta* = −0.37, Cohen’s *d* = 0.80, *p =* 0.020), yet not “mild pain” (*beta* = 0.01, Cohen’s *d* = 0.02, *p =* 0.966) or “moderate pain” (*beta* = 0.01, Cohen’s *d* = 0.02, *p =* 0.942) (Fig. 1). Within the AD group, temperatures required to reach each target percept and pain unpleasantness values were not meaningfully associated with depressive symptoms (*r* = 0.06–0.22, *p* > 0.20). Thus, analyses controlling for depressive symptoms did not modify the reported findings. Consistent with the between-groups analyses, no statistically significant associations of worsening global cognitive function were observed with ratings of unpleasantness within the AD group: warmth: *beta* = −0.22, Cohen’s *d* = 0.45, *p* = 0.167; mild pain: *beta* = −0.20, Cohen’s *d* = 0.41, *p* = 0.221; moderate pain: *beta* = −0.21, Cohen’s *d* = 0.43, *p* = 0.192 (Fig. 2).

## Discussion

This study compared the perception threshold for three experimental heat pain intensities and reports of unpleasantness associated with each of the three percepts in a sex and age-matched sample of older adults with and without mild to moderate cognitive impairment in AD. Our first hypothesis, that people with AD would be less sensitive to thermal pain and find pain to be less unpleasant than would cognitively intact controls, was partially supported. Compared to healthy controls, people diagnosed with AD were less pain sensitive: they demonstrated higher intensity thresholds for the detection of “warmth,” “mild pain,” and “moderate pain.” However, despite requiring higher stimulus temperatures to reach these three perceptual thresholds, people with AD reported similar levels of pain-associated unpleasantness across the three percepts when compared to controls. Our second hypothesis, that as global cognitive function worsens, people with AD would become less sensitive to pain and find pain less unpleasant, was not supported.

Our findings are in agreement with several previous studies. Cole and colleagues [17] found that, compared to controls, people with AD demonstrated higher mechanical pressure pain thresholds (less sensitivity) for just-noticeable pain and weak pain. Similarly, Gibson and colleagues [15] found that, compared to controls, people with AD demonstrated increased detection thresholds for pain sensation.

Unlike many previous studies, the current study also examined self-reports of unpleasantness ratings associated with each perceptual threshold examined (“warmth,” “mild pain,” and “moderate pain”) and found no differences in unpleasantness ratings between controls and participants with AD. Finding no differences in self-reported pain unpleasantness between controls and people with AD initially seems to be in contrast to work demonstrating increased facial affective pain responses in AD [5, 14, 19]. Although reasons cannot be conclusively determined, these differences across studies may relate to the psychophysical methods used in the current study (the stimulus intensity of each percept level was individually adjusted for each individual rather than using a fixed intensity) or in the measures used to assess the affective response to pain (self-report ratings versus observed facial affect).

Interestingly, when examining the association between global cognitive function and pain responses, the current study found no evidence to suggest that worsening global cognitive function was associated with lower reports of unpleasantness in the presence of mild and moderate pain. This finding does not appear to support a longstanding hypothesis that the affective/motivational component of pain may be altered in AD [36]. The current findings appear somewhat divergent with findings of Benedetti and colleagues [16], who reported that tolerance to highly unpleasant evoked pain stimuli (electrical shock and ischemic arm pain) was increased in people with worsening AD. Furthermore, Scherder and colleagues [37] found that, compared with controls, people with AD reported lower pain intensity and pain affect on repeated daily pain assessments. Similarly, an earlier study by Scherder and colleagues [38] found that, after matching for painful conditions and despite receiving similar amounts of analgesics, people with AD reported lower pain intensity and pain affect than non-AD controls [38]. In addition to using a perceptually matched psychophysics paradigm in the current study, a likely reason for differences in unpleasantness ratings between previous and current study findings is that Scherder and colleagues [38] used a 5-item number of chosen words-affective scale [39] versus a 0–20 unpleasantness scale such as that used in the current study.

Taken together with previous work, findings from the current study continue to demonstrate that, when compared to healthy individuals, people with AD seem to have altered detection levels for painful stimuli. The current findings seem to support an emerging pattern in the literature demonstrating that detection thresholds for lower, less noxious, levels of pain may be elevated in individuals with AD compared to those without AD. In contrast, although the current study found increased thresholds for detecting moderate pain in people with AD as a group compared to non-AD controls, there did not appear to be any influence of worsening global cognitive impairment on detection threshold for moderate pain (effect sizes were near zero). Thus, current findings partially support and extend previous work by Scherder and colleagues [37], demonstrating that people with AD may be less sensitive to pain than healthy controls. However, we found no evidence using controlled evoked pain stimuli to corroborate their findings that clinical pain becomes less unpleasant as AD severity worsens.

The clinical implications of altered reporting of pain in AD patients may include increased risk of late or failed detection of an underlying pathology requiring immediate attention. Diminished ability to detect pain could lead to increased negative outcomes once pain is recognized. For example, Morrison and colleagues found that when assessing post-operative hip pain, people with AD reported increased levels of pain, increased length of hospitalization, and decreased ability to ambulate 3 months post-operatively [3].

There are some limitations in the current study that should be considered when interpreting results. People with an existing clinical diagnosis of AD from several different clinician practices were included and thus no single AD diagnostic procedure was selected. We realize that other sensory modalities (e.g., somatosensory, auditory) may have contributed to the overall pain experience; however, we believe the current study design examining pain psychophysics helps to address a clinically relevant problem, altered pain processing in AD. Interestingly, we did not find an association between perceptual pain thresholds and depression in the AD group. We believe, however, it is possible that the limited variability of GDS-SF scores within the AD group contributed to the failure to uncover an association between depression and pain. A perceptual matching paradigm was used for thermal sensory detection levels. Because of this, individual ratings of unpleasantness were based on perceptually matched thresholds for warmth, mild pain, and moderate pain and not on a fixed temperature paradigm. This procedure may have impacted on the pattern of findings for pain unpleasantness in unknown ways. Despite these limitations the current study adds to the limited number of clinical and experimental pain studies examining altered pain response in AD.

Distinguishing between pain intensity and pain affect in both the acute and chronic pain context is critical to the translational relevance of future pain studies in dementia. Although the literature continues to demonstrate mixed results, it is becoming increasingly clear that, relative to non-AD controls, people with AD generally have an altered response to clinical and experimental pain. It has been suggested that clinicians consider using both intensity and affective (qualitative) scales when assessing pain in AD [38]. Moreover, as recently demonstrated by several laboratories [5, 14, 19], assessing nonverbal emotion-related measures such as facial affect during acutely painful experiences (e.g., bathing, turning, trauma, post-operative procedures) may be particularly useful in the AD population.

## Conclusions

Findings from the current study support the idea that the “pain threshold” may have less relevance to clinical pain states, and that “suprathreshold” pain responses need to be examined in a systematic fashion. Moving forward, it is critical to increase our understanding of the psychophysical response to pain in dementia, yet in order to complement this important work, we urgently need to increase our understanding of the underlying neurophysiological alterations contributing to pain alterations in dementia. As people age, the risk of developing pain increases and as the population of older adults continues to grow, so will the number of people diagnosed with dementia who have pain. Finding ways to improve pain care in people with all forms of dementia is crucial to alleviate unnecessary suffering in this highly vulnerable population.

### Availability of data and materials

The original data used in the preparation of this manuscript are presented in Additional file 1.

## Additional file

Additional file 1: Psychophysics Raw Data. (XLSX 17 KB)
